# Primary Central Nervous System Vasculitis: A Rare Cause of Stroke

**DOI:** 10.7759/cureus.39541

**Published:** 2023-05-26

**Authors:** Ana de Carmo Campos, Sara Sarmento, Marco Narciso, Teresa Fonseca

**Affiliations:** 1 Pulido Valente Hospital, North Lisbon University Hospital Centre, Lisbon, PRT; 2 Department of Health Promotion and Prevention of Non-communicable Disease, National Health Institute Doutor Ricardo Jorge, Lisbon, PRT; 3 Faculty of Medicine, University of Lisbon, Lisbon, PRT

**Keywords:** brain ultrasonography, magnetic resonance angiography, primary central nervous system vasculitis, ischemic stroke, angiitis

## Abstract

Primary central nervous system vasculitis (PCNSV) is a rare cause of ischemic stroke and is considered idiopathic in most cases. PCNSV can present with a wide variety of neurological manifestations and should be considered in the differential diagnosis of ischemic stroke, particularly if the neurological deficit cannot be explained by the affected vascular area or when it is multifocal. The diagnosis of PCNSV is relevant because the required therapy differs from the treatments commonly used for frequent ischemic strokes.

We report the case of a 64-year-old woman admitted for an ischemic stroke with a right frontal cortico-subcortical ischemic lesion. The etiological investigation documented multiple intracranial arterial stenoses. Secondary causes of central nervous system vasculitis were excluded. The patient refused a brain biopsy, and corticosteroid therapy was initiated due to high suspicion of PCNSV, supported by findings from transcranial Doppler ultrasound and brain magnetic resonance angiography. The patient had a positive clinical outcome and did not have any recurrences while under therapy.

This case raises awareness of the importance of considering PCNSV in the differential diagnosis of ischemic stroke. It emphasizes the importance of promptly initiating therapy to minimize PCNSV-associated complications.

## Introduction

Primary central nervous system vasculitis (PCNSV) (or angiitis) is a rare disease with an incidence of 2.4 cases/1 million people/year, and its pathophysiology remains unclear in most cases [1,2]. It is characterized by an inflammation of small and medium cerebral vessels without systemic involvement and can occur at any age.

PCNSV can present a wide variety of neurological manifestations, making it a diagnostic challenge [2-5]. Clinically and radiologically, PCNSV may mimic other entities, with reversible cerebral vasoconstriction syndrome (RCVS) standing out [6]. The treatment of PCNSV is distinct from others used for frequent ischemic strokes, and its early diagnosis is important for the institution of adequate therapy. It should be considered in the etiological study of ischemic stroke, particularly if the neurological deficit is not explained by the affected vascular area or if the involvement is multifocal [2,4,7].

We present the case of a patient with an ischemic stroke due to PCNSV with a good clinical outcome with corticosteroid therapy.

## Case presentation

A 64-year-old woman, without any known vascular risk factors, presented to the Emergency Department (ED) due to left hemiparesis and dysarthria with approximately two hours of evolution. The patient reported previous self-limiting and recurring episodes of left hemiparesis over the past three days. She also experienced a mild holocranial headache, without any other associated symptoms, namely, involuntary movements, loss of consciousness, or mood changes.

On clinical observation, she exhibited mild dysarthria and left hemiparesis with brachial predominance (muscle strength grade 4/5 left upper limb and 4+/5 left lower limb), without other focal neurological deficits (National Institutes of Health Stroke Scale, NIHSS 3). No fever or any other noteworthy findings were noted during the evaluation.

**Figure 1:**
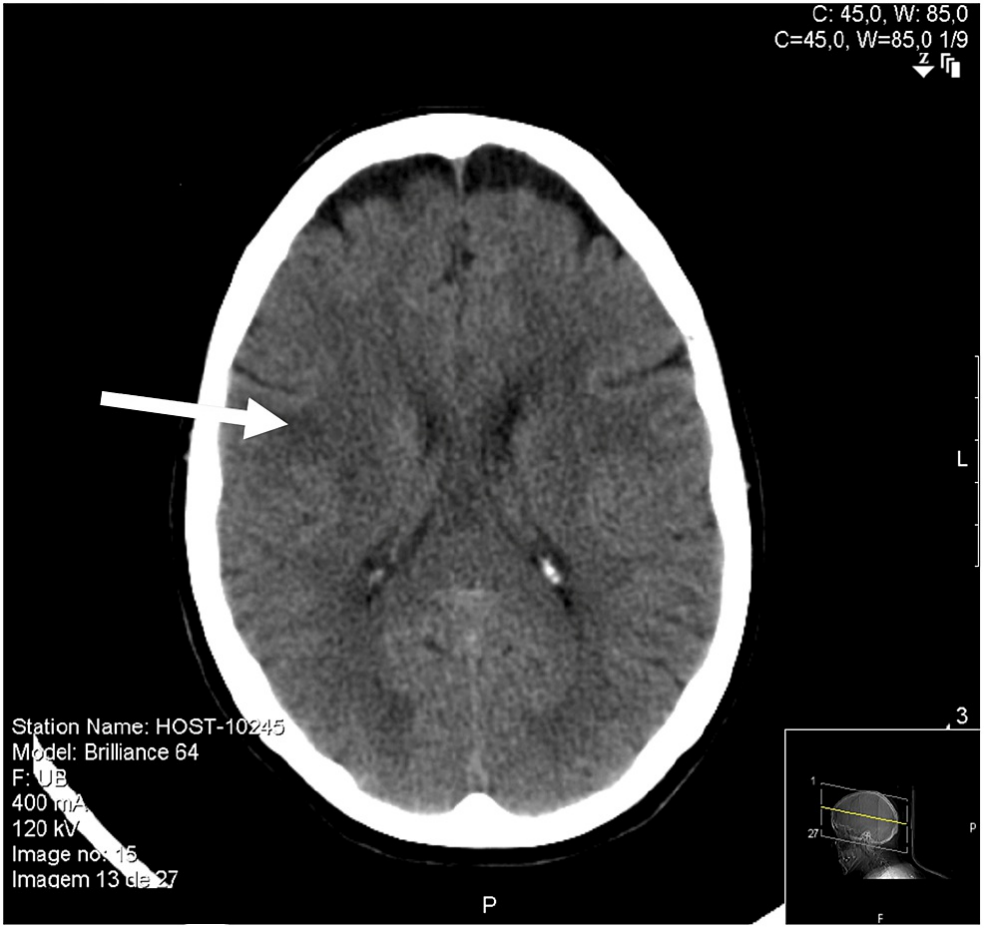
Brain computed tomography performed in the emergency department reveals right frontal cortico-subcortical hypodensity (arrow).

**Figure 2:**
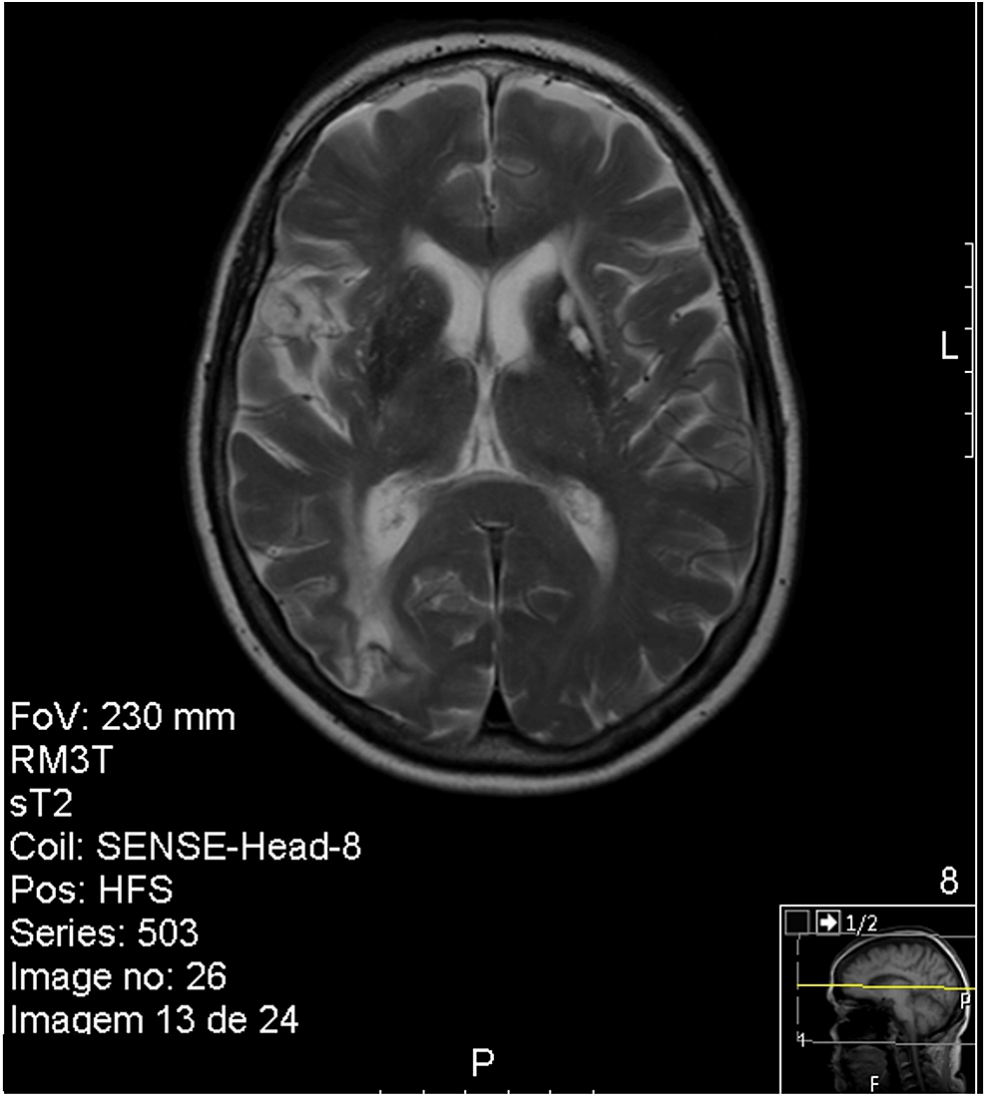
Brain magnetic resonance imaging (FLAIR) reveals multiple hyperintense cortico-subcortical lesions, frontal, parietal, and in the right occipital temporal transition. FLAIR: fluid-attenuated inversion recovery

**Figure 3:**
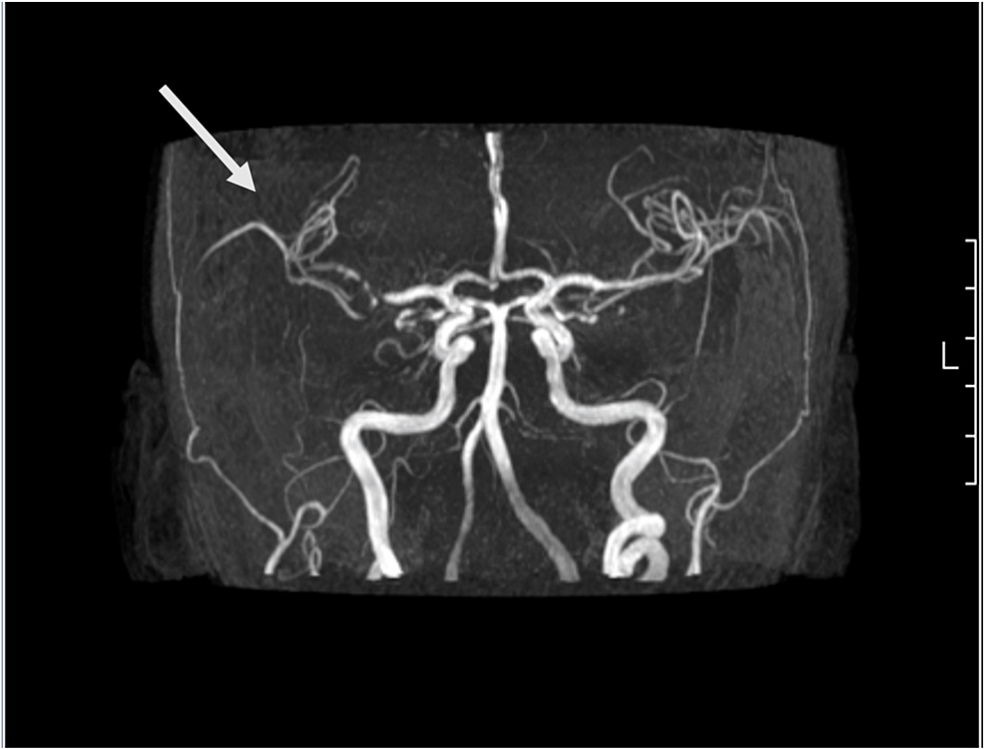
Brain magnetic resonance angiography on day six of hospitalization showing multiple areas of stenosis most pronounced in the intracranial arterial system, specifically in the M1 and M2 segment of the right MCA (arrow), as well as stenosis at superior and anteroinferior cerebellar arteries. MCA: middle cerebral artery

Computed tomography angiography (CTA) was performed at the ED showing a right frontal cortico-subcortical ischemic lesion (Figure 1). The patient was hospitalized due to a right hemisphere stroke, without indication for reperfusion therapy (NIHSS 3 and absence of large vessel occlusion). The patient began treatment with antiplatelet therapy and high-dose statin therapy. Doppler ultrasonography of the neck vessels was performed, with no relevant changes, namely, without atheromatous plaques. A transcranial Doppler ultrasound revealed a significant increase in flow velocity in the distal segment of the right middle cerebral artery (MCA) (systolic velocity (SV) 470 cm/second; diastolic velocity (DV) 225 cm/second), the right posterior cerebral artery (PCA) (SV 300 cm/second; DV 95 cm/second), and the left MCA and PCA (SV 170 cm/second; DV 50 cm/second), suggestive of multiple and important arterial stenoses. Brain magnetic resonance imaging (MRI) showed several hyperintense, cortico-subcortical, frontal, parietal, and right occipital temporal transition lesions (Figure 2). Brain magnetic resonance angiography (MRA), performed with the vessel wall protocol, revealed multiple areas of stenosis in the intracranial arterial system, suggesting vasculitis. These stenoses were observed in the M1 and M2 segments of the right MCA, distal branches of the left MCA, as well as in the superior and anterior inferior cerebellar arteries (Figure 3). No abnormalities were detected in the electrocardiogram, 24-hour Holter monitoring, and transthoracic echocardiogram with Doppler study.

Laboratory results showed blood count with an unchanged leukogram and normal platelet count (243 × 10^9^/L, normal range: 150-450 × 10^9^/L), as well as slight elevation of the erythrocyte sedimentation rate (27.0 mm/hour, normal range: <15 mm/hour) and of the C-reactive protein (0.99 mg/dL, normal range: <0.5 mg/dL). No changes in lipid profile and thyroid function were noted. No abnormalities were found in the hemostasis study, and the autoimmune study yielded negative results (Table 1). The serology tests for hepatitis (HBV and HCV) and retroviruses (HIV 1 and HIV 2) were negative, along with negative results for *Treponema pallidum* antibodies and *Mycobacteria *testing. The cerebrospinal fluid (CSF) showed no cytochemical alterations, with negative results for herpes viruses (herpes simplex type 1 and type 2 and varicella-zoster), *Treponema pallidum*, cytomegalovirus, Epstein-Barr virus, and *Borrelia burgdorferi* (Table 2). No microbiological agents were isolated in the blood and CSF cultures.

**Table 1:** Main laboratory parameters in blood tests. c-ANCA: cytoplasmic antineutrophil cytoplasmic autoantibody; dsDNA: anti-double stranded DNA antibodies; ESR: erythrocyte sedimentation rate; HBV: hepatitis B virus; HCV: hepatitis C virus; HIV: human immunodeficiency virus; HSP: heat shock protein 70; JO1: antibody-positive myositis; MPO: myeloperoxidase; RR: reference range; Sm: anti-Smith antibody; SnRNP: small nuclear ribonucleoprotein; SSA: anti–Sjögren’s-syndrome-related antigen type A; SSB: anti–Sjögren’s-syndrome-related antigen type B ^1^:ESR reference range: <15 mm/hour; ^2^: Reactive C-Protein reference range: <0.5 mg/dL.

Laboratory parameters	Result
ESR	27 mm/hour^1^
C-reactive protein	0.99 mg/dL^2^
Activated partial thromboplastin time (patient/control)	30/31 seconds
Prothrombin time (patient/control)	11.2/11.6 seconds
Lupus anticoagulant	Negative
Anticardiolipin	Negative
Anti-beta 2 glycoprotein 1 antibody	Negative
Anti-dsDNA antibody	Negative
Antinuclear antibodies (ANA)	Negative
Anti-cytoplasm antibodies (c-ANCA)	Negative
Anti-MPO antibodies	Negative
SSA antibodies	Negative
SSB antibodies	Negative
Sm antibodies	Negative
SnRNP antibodies	Negative
HSP70 antibodies	Negative
JO1 antibodies	Negative
Complement (C3, C4)	No consumption
Protein electrophoresis	No changes
Hepatitis serology (HBV, HCV)	Negative
Retroviruses (HIV 1, HIV 2)	Negative
*Treponema pallidum* antibodies	Unreactive
Mycobacterial culture	Negative

**Table 2:** Main laboratory parameters in cerebrospinal fluid analysis. HBV: hepatitis B virus; HCV: hepatitis C virus; HIV: human immunodeficiency virus; PCR: polymerase chain reaction

Laboratory parameters	Result
Cytochemical and biochemical examination	No changes
Hepatitis serology (HBV, HCV)	Negative
Retroviruses (HIV 1, HIV 2)	Negative
Herpes viruses (herpes simplex type 1 and type 2, and varicella-zoster)	Negative
Epstein-Barr virus	Negative
Borrelia burgdorferi	Negative
Cytomegalovirus	Negative
*Treponema pallidum* antibodies	Unreactive
Mycobacteria testing (PCR amplification)	Negative

Due to a high suspicion of PCNSV, corticosteroid therapy was initiated at a dosage of 1 mg/kg of body weight. The patient showed good clinical progress and improvement in the degree of stenosis, which was reevaluated using transcranial Doppler ultrasonography 10 days after initiating treatment. This follow-up examination revealed a substantial decrease in flow velocities, especially in MCA (Table 3). The patient refused a brain biopsy and was discharged under corticosteroid therapy (NIHSS 2, mRankin 1) to be reevaluated as an outpatient.

**Table 3:** Evolution of cerebral artery flow velocities evaluated by transcranial Doppler ultrasonography. D: day; CT: corticosteroid therapy; MCA: middle cerebral artery; PCA: posterior cerebral artery; SV: systolic velocity; DV: diastolic velocity; V_peak_: peak systolic velocity; V_mean_: mean velocity (average of the peak systolic and peak diastolic components) Normal mean flow velocity in MCA 55 ± 12 cm/second; stenosis: mild >120 cm/second; moderate >160 cm/second; severe >200 cm/second. Normal mean flow velocity in PCA 34 ± 8 cm/second.

Vascular territory	Transcranial Doppler ultrasound examinations	Flow velocities (cm/second)	
		V_peak_	V_mean_
Right MCA	Admission	470	347.5
	D1 CT	370	250
	D10 CT	285	232
Right PCA	Admission	300	197.5
	D1 CT	270	172.5
	D10 CT	250	162.5
Left MCA and PCA	Admission	170	110
	D1 CT	170	95
	D10 CT	150	100

The patient was readmitted six months after being discharged, presenting with an ischemic stroke in the same vascular territory and exhibiting similar clinical symptoms of left hemiparesis and dysarthria. Subsequently, it was discovered that the patient did not attend follow-up appointments after being discharged and independently discontinued corticosteroid therapy due to cushingoid appearance. The reintroduction of corticosteroid therapy led to an improvement in the patient’s condition, and no relapses have been reported to date.

## Discussion

The diagnostic criteria for PCNSV were initially proposed by Calabrese and Mallek (1988) and included the following: (a) the presence of unexplained neurological deficit after thorough clinical and laboratory evaluation; (b) documentation by cerebral angiography and/or biopsy revealing arteritis in the CNS; and (c) and the absence of systemic vasculitis or other secondary condition associated with the angiographic or pathological features described [8]. Currently, working groups are looking for new criteria and methods for the diagnosis of PCNSV, as well as the best therapeutic strategy [4,7,9,10].

Brain biopsy remains the gold standard in the diagnosis of PCNSV, but patchy or inaccessible lesion involvement may result in false negatives [11-13]. Angiography is considered the gold standard in the imaging diagnosis of PCNSV. Currently, a diagnostic sensitivity of 90-100% is recognized for brain MRA, and its value in the diagnosis of angiitis is increasing [10,14,15].

Our patient revealed a focal neurological deficit, and the initial imaging findings were suggestive of an ischemic stroke. Vascular occlusive causes of stroke were ruled out in the patient with no other identified vascular risk factors, except for stage 1 hypertension which was diagnosed and treated during hospitalization.

The presence of multiple stenoses across different vascular territories observed on brain imaging, along with the findings from transcranial Doppler ultrasound, strongly indicated the presence of vasculitis. Secondary causes of CNS vasculitis such as infections, systemic vasculitis, autoimmune diseases, and/or lymphoproliferative diseases were excluded [3]. The combination of unexplained recurrent neurological deficits, angiographic features indicative of CNS angiitis, and the absence of systemic vasculitis are established diagnostic criteria for PCNSV [8].

Because a brain biopsy was not performed, histological information regarding an inflammatory process associated with vasculitis could not be obtained. Therefore, the possibility of RVCS cannot be completely ruled out. This condition is equally rare and shares similar clinical and imaging features with PCNSV [16].

In RVCS, the primary manifestation is a sudden and intense thunderclap-like headache. The clinical presentation of RVCS can vary widely in terms of severity and may be accompanied by hypertension and focal neurological deficits. The etiopathogenesis of RVCS in relation to ischemic stroke remains unclear [6]. Histopathological studies in patients with RCVS document minimal vascular changes, pointing to functional vasoconstriction rather than a vasculitis phenomenon characteristic of PCNSV, highlighting the increasing role of brain MRA as a noninvasive diagnostic option [14,15].

The diagnosis of PCNSV holds importance due to its unique therapeutic approach, which differs from the management of other common causes of ischemic stroke. This approach typically involves the administration of systemic glucocorticoids, with a lower risk of symptom recurrence if used in combination with cyclophosphamide [4,9,10]. While in PCNSV there is a favorable response to corticosteroid therapy, it should be avoided in RCVS because its use is considered an independent predictor of a worse prognosis [6].

Our patient had no other known vascular risk factors and no signs of atheromatosis in the cervical vessels. In addition to neurological deficits, she presented with symptoms of a mild-to-moderate and self-limiting holocranial headache. Imaging studies revealed the presence of multiple intracranial stenoses, which were suggestive of vasculitis. Finally, the patient exhibited a positive response to corticosteroid therapy, with a favorable clinical outcome. Therefore, PCNSV was considered the underlying cause of the ischemic stroke, rather than RVCS.

## Conclusions

There are few described cases of PCNSV, and its presentation can mimic other forms of ischemic stroke, making it a diagnostic challenge. Given its high sensitivity, brain MRA may become an alternative to invasive diagnostic techniques. Transcranial Doppler ultrasound plays a significant role when there is initial suspicion of PCNSV as it can reveal hemodynamic changes associated with vasculitis.

Early diagnosis of PCNSV enables prompt initiation of therapy, minimizing complications related to recurrent vasospasm. This leads to a favorable clinical course and prognosis. Further research is necessary to investigate therapeutic strategies for PCNSV, including optimal treatment duration and potential combination with other immunosuppressive agents. Additionally, exploring new therapeutic targets that are free from the known side effects of corticosteroids is crucial to enhance treatment adherence and effectiveness.
